# En route to single-step, two-phase purification of carbon nanotubes facilitated by high-throughput spectroscopy

**DOI:** 10.1038/s41598-021-89839-4

**Published:** 2021-05-19

**Authors:** Blazej Podlesny, Barbara Olszewska, Zvi Yaari, Prakrit V. Jena, Gregory Ghahramani, Ron Feiner, Daniel A. Heller, Dawid Janas

**Affiliations:** 1 Department of Organic Chemistry, Bioorganic Chemistry and Biotechnology, Silesian University of Technology, B. Krzywoustego 4, 44-100 Gliwice, Poland; 2 Molecular Pharmacology Program, Memorial Sloan Kettering Cancer Center, New York, NY USA; 3 Department of Pharmacology, Weill Cornell Medicine, New York, NY USA

**Keywords:** Carbon nanotubes and fullerenes, Nanophotonics and plasmonics

## Abstract

Chirality purification of single-walled carbon nanotubes (SWCNTs) is desirable for applications in many fields, but general utility is currently hampered by low throughput. We discovered a method to obtain single-chirality SWCNT enrichment by the aqueous two-phase extraction (ATPE) method in a single step. To achieve appropriate resolution, a biphasic system of non-ionic tri-block copolymer surfactant is varied with an ionic surfactant. A nearly-monochiral fraction of SWCNTs can then be harvested from the top phase. We also found, via high-throughput, near-infrared excitation-emission photoluminescence spectroscopy, that the parameter space of ATPE can be mapped to probe the mechanics of the separation process. Finally, we found that optimized conditions can be used for sorting of SWCNTs wrapped with ssDNA as well. Elimination of the need for surfactant exchange and simplicity of the separation process make the approach promising for high-yield generation of purified single-chirality SWCNT preparations.

## Introduction

Single-walled carbon nanotubes (SWCNTs)^1^ exhibit extraordinary electrical^2,3^, thermal^4,5^, mechanical^6,7^ and optical^8–11^ characteristics. Despite their unique properties, difficulties with purification methods have hampered their utility for many applications. For instance, for electronic and mechanical devices^12^, control over the chirality of SWCNTs is important. Unfortunately, synthesis of this carbon allotrope gives mixtures of various CNT types of drastically different properties from one another^13^. Due to the presence of multiple species in the unsorted material, the overall performance of their ensembles are far from expectations^14,15^. Nevertheless, purified CNT dispersions are considered very promising for applications in various fields. In photonics, for example, they offer high performance as single photon emission sources^16^. In the biomedical fields, single-chirality purification of SWCNTs enables improvements in performance and biocompatibility for applications in imaging and optical biosensors^17–19^. It is clear that single-chirality purification of SWCNTs is therefore desirable to improve the utility of this class of materials^20^.

Recently, an arsenal of methods was established to tackle the problem of CNT sorting^13^. CNT dispersions can now be enriched with species of appropriate electrical character^21,22^, (n,m) species^23–25^ or even handedness^26^ by procedures such as electrophoresis^27^, density gradient ultracentrifugation (DGU)^28^, chromatography^29,30^, selective solubilization with polymers^31–33^ or biphasic extraction^23,34–37^. Specifically, a variation of the former technique, the aqueous two-phase extraction (ATPE) revealed a notable potential for this purpose because of a range of advantage that it offers. It is simple, quick, scalable, based on common laboratory chemical compounds and, most importantly, enables high precision separation of CNTs both in the small-^35,36,38^ and large-diameter regimes^24^. In this process, stepwise iterative separation of CNTs distributed between two phases enables their fractionation. Most of the reported routines are of multi-step nature and targeted at resolution of all the components of the parent CNT mixture. We^21,25^ and others^38^ have recently started exploring the concept of single-step separation of CNTs by means of ATPE to obtain only the desired CNT types using the simplest possible methods. Such separation requires redox-^21,37^ or pH-modulating^25,38^ chemical compounds to enhance the otherwise minute differences in how various CNT types are hydrated to enable separation at high resolution.

In this contribution, we present efforts to effect single-step purification of individual SWCNT chiralities, and techniques to monitor this separation by high-throughput photoluminescence spectroscopy. Thorough analysis of the parameter space of the ATPE enabled careful tuning of conditions to obtain SWCNTs fractions of a single chirality. The method employs ionic and non-ionic surfactants, to modulate the partitioning process of the SWCNTs between phases. Importantly, we were able to employ the optimized parameters for surfactant wrapped SWCNTs to sort SWCNTs dispersed with DNA as well. This process eliminated the need for an surfactant exchange processing steps. This work has implications for the scale-up of SWCNT purification efforts, and it demonstrates a new role that near-infrared spectroscopy may play in the benchmarking of these processes.

## Experimental

### Chemical compounds

Dextran (DEX, MW ca. 250,000 Da, Alfa Aesar), poly(ethylene glycol) (PEG, MW ca. 6000 Da, Alfa Aesar), sodium cholate hydrate (SC, Sigma-Aldrich), Pluronic F-127 (PL, Sigma-Aldrich), hydrochloric acid (HCl, Sigma-Aldrich) and sodium hydroxide (NaOH, Sigma-Aldrich) were all obtained from the shown vendors and had pure p.a. class. The ssDNA sequence TTT-CCC-TTT-CCC-CCC and the buffer IDTE (10 mM Tris, 0.1 mM EDTA, pH 7.5) were acquired from Integrated DNA Technologies.

For this work, two types of SWCNTs prepared by the CoMoCAT process were used. Unsorted (EG150x) and (6,5)-enriched materials, both of which were procured from CHASM Advanced Materials (Canton, MA, USA).

In all cases when a solvent was used for sorting or characterization, it was nuclease-free water for RNA work (Fisher Bioreagents) to keep the environment of the biphasic system as consistent as possible, but, most importantly, to guarantee compatibility of the medium with DNA dispersed CNTs.

### Preparation of SWCNT dispersions

SWCNT powder (10 mg) was introduced to a 2 wt% sodium cholate solution in water (10 mL). Where indicated, the aforementioned DNA sequence was used as a dispersing agent instead. In this case, (6,5)-enriched SWCNTs were mixed with ssDNA (10 mg/mL in IDTE buffer) at a 2:1 (w/w) in 1 mL of phosphate buffered saline (PBS) and sonicated for 30 min at 40% amplitude (Sonics & Materials). Following sonication, the samples were ultracentrifuged (Sorvall Discovery 90SE) at 250,000 × *g* for 40 min. The top 90% of the supernatant was kept. To remove free ssDNA, 100 kDa Amicon centrifuge filters (Millipore) were used. The ssDNA wrapped SWCNTs were re-suspended in PBS.

For both approaches, the CNT-dispersant mixture was homogenized with an ultrasound tip sonicator (Sonics & Materials) for 30 min with a constant power of 50 W. The mixture was kept in an ice bath during sonication to ensure proper dispersion of the material. After sonication, the dispersion was immediately centrifuged (Eppendorf Centrifuge 5430 R) at constant temperature (18 °C) at the relative centrifugal force of 30,000 × *g* for 2 h. The upper 80% of supernatant was collected for experiments.

### ATPE protocol

Aqueous solutions of PEG, DEX, SC (concentrations of 50 wt%, 20 wt%, and 10 wt%, respectively) and Pluronic (concentration of 10 wt%) were transferred in the specified order to a centrifuge tube (2 mL) and homogenized by a vortex mixer for 10 s. Then, where indicated, selected volumes of 0.1 M HCl or 0.1 M NaOH were introduced at this point and vortexed for 10 s. Finally, the CNT dispersion was added and the sample was homogenized again by vortexing for time specified above. The resulting cloudy suspensions were centrifuged at a constant temperature (18 °C) at the relative centrifugal force of 2025 × *g* for 3 min to facilitate phase separation. After the centrifugation, resulting two phases were immediately collected by pipetting. In each experiment, the total volume of the ATPE system was 1.530 mL. Exact parameters of the separation routines are provided in the manuscript or accompany the “Supplementary files”.

### Optical characterization

Photoluminescence excitation-emission (PLE) maps were acquired using a custom-built setup to conduct high-throughput spectroscopy on SWCNT samples in a well plate^39^. Briefly, the sample, located in a 96 well plate on an automated stage on an Olympus IX-71 microscope, was excited through a 20× microscope objective using a supercontinuum laser (NKT SuperK Extreme EXR15) coupled to a variable bandpass filter (NKT SuperK Varia High) to tune the excitation from 500 to 827 nm, in 3 nm steps, with a 20 nm bandwidth. The resulting emission was collected through the same objective and directed into a spectrometer (Princeton Instruments IsoPlane SCT 320), with a focal length of 320 mm and an aperture ratio of f/4.6, which was coupled to a TE-cooled InGaAs array detector (Princeton Instruments 640 × 512 pixel NIRvana: 640) with a 20 μm pixel size, and a quantum efficiency of > 85% in the detection range of 0.9–1.7 μm. The acquisition time was 1 s for each spectrum, resulting in a full PLE plot in approximately 2.5 min registered in a fully automated fashion. Therefore, numerous samples can be analyzed per day. Corrections were applied for wavelength-dependent variations in excitation power (5–30 mW measured at the sample), as well as grating and detector efficiencies. PLE maps were normalized to the optical feature of the highest intensity. UV–Vis-NIR spectra were taken using a V-670 JASCO spectrophotometer. Raman spectra (Renishaw RM1000) were acquired in the Radial Breathing Mode (RBM) area using λ = 633 nm laser at 10% power. Multiple spectra at the integration time of 10 s were recorded to increase the signal to noise ratio.

## Results and discussion

### Analysis of starting material

**Figure 1:**
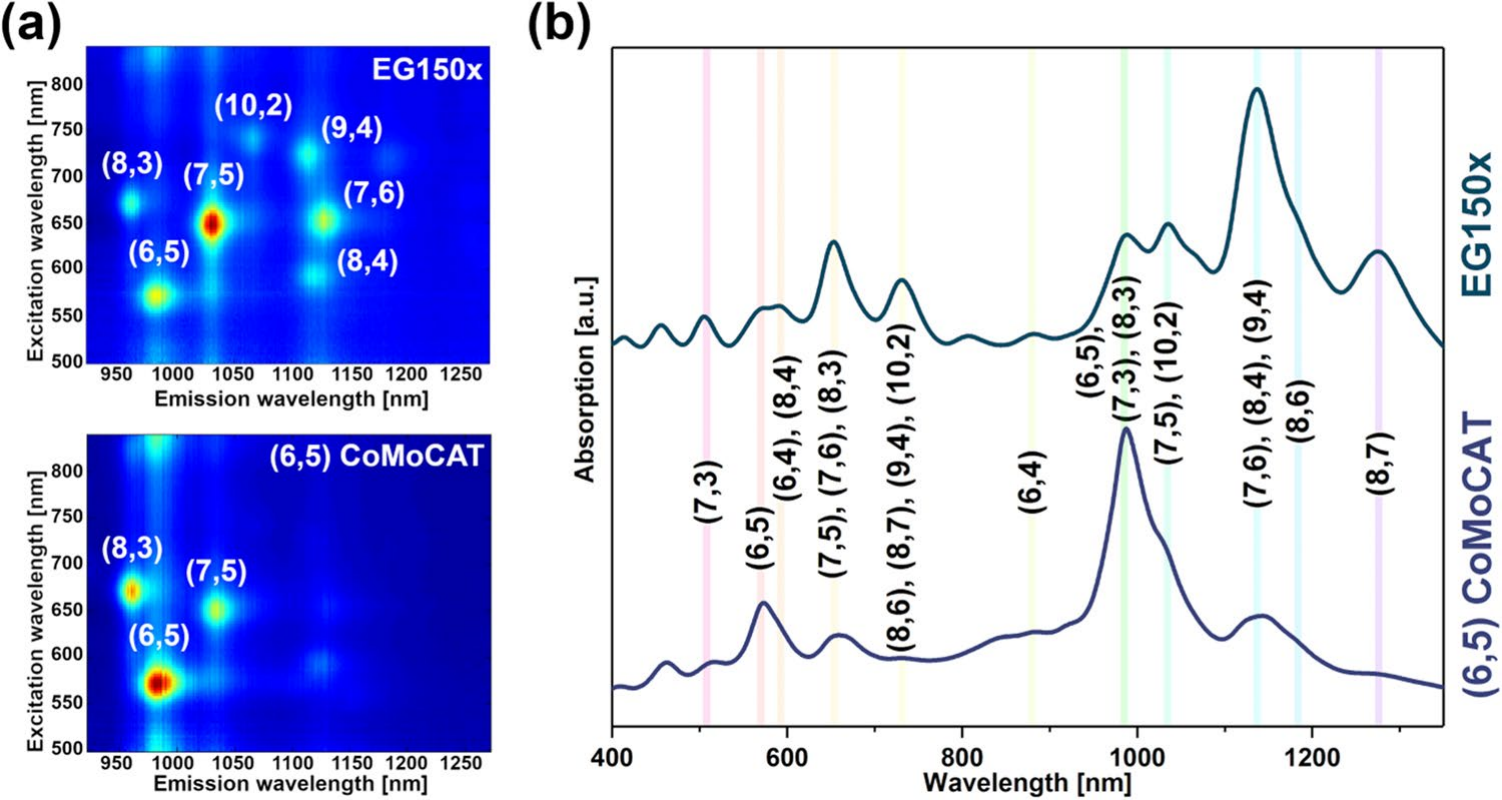
(**a**) 2D PLE maps of unsorted and (6,5)-enriched SWCNT materials dispersed in aqueous SC solution. (**b**) Corresponding optical absorption spectra.

Optical characterization of two parent materials selected for the study, EG150x and (6,5)-enriched CoMoCAT preparations, by 2D PLE mapping and absorption spectroscopy, revealed the presence of multiple CNT types (Fig. 1).

The unsorted (EG150x) material was composed of diverse chiralities, out of which seven manifested distinct signatures when measured using rapid acquisition of PLE maps (Fig. 1a) (approximately 2.5 min each). On the other hand, the (6,5)-enriched material contained a reduced number of species as expected. It was comprised predominantly of CNTs of (6,5) type, but (8,3) and (7,5) were also detected (Fig. 1b). Corresponding peaks of SWCNTs discerned by PLE were found in the absorption spectra, which presents the E_11_ and E_22_ transitions (Fig. 1c). Additional peaks shown in the absorption spectra originate from SWCNT species of metallic character or semiconducting SWCNTs present typically at low concentrations^26,40,41^.

### Influence of ionic surfactant

**Figure 2:**
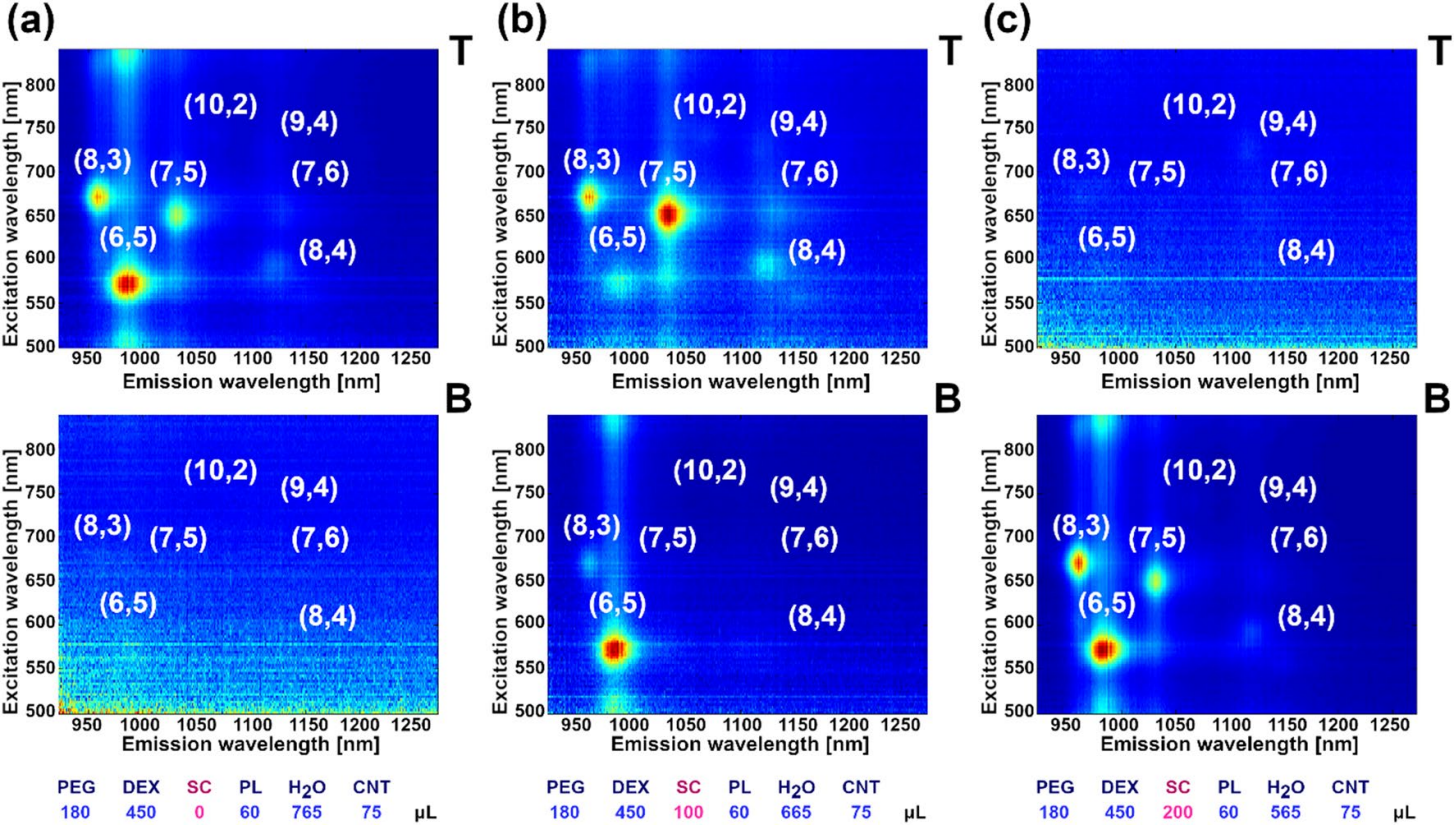
2D PLE maps of SC-dispersed, (6,5)-enriched SWCNTs sorted by ATPE upon varying the added volume of SC. Example volumes: (**a**) 0 µL, (**b**) 100 µL and (**c**) 200 µL of SC. 2D PLE maps of remaining samples are shown in Supplementary Animation 1. Analogous data for unsorted SWCNT material is presented in Fig. S2 and Supplementary Animation 2.

Separation of SWCNTs by ATPE utilizes competition of surfactants for adsorption on the SWCNT surface^42^. Depending on the structure and electronic properties of the SWCNTs, deposition of particular surfactant molecules on certain SWCNT species is more favorable. The better a CNT is encapsulated, the better the hydrophobic SWCNT nature is cloaked inside the micelles. Differences in the hydrophilic/hydrophobic character, and the affinity of a surfactant@SWCNT hybrid towards one of these media, directs it to the top or to the bottom phase. In case of SC, the shift generally takes place from a more hydrophobic top phase composed of PEG to more hydrophilic bottom phase made of dextran (Fig. 2)^42^.

We observed that for the selected separation parameters, initially all SWCNTs remained in the top phase (Fig. 2a). Stepwise increases in the concentration of SC gradually moved the SWCNTs to the bottom phase in the following order: (6,5), (8,3), then (7,5) (Fig. 2b,c). We note that a diameter cut-off mechanism is valid herein, since these species are of 0.757, 0.782 and 0.829 nm diameters, respectively.

Interestingly, while analyzing the process in greater detail, we observed that the transition from one phase to the other was rapid and occurred once a defined concentration threshold of SC was reached. A similar observation was recently noted in parallel by Sims and Fagan, who investigated the mechanics of ATPE separation by varying the SDS content^43^. In the present work, the addition of 95 µL, 106 µL and 134 µL of SC corresponded with the maximum rate of partitioning (Fig. S1, Supplementary Animation 1) from the top to the bottom. At this point, half of the SWCNT species moved to the complementary phase. Modulation of SC alone was insufficient to produce (6,5)-enriched material, as transition curves for (6,5), (8,3) and (7,5) SWCNTs overlapped (please refer to the top panels of the “Supplementary Animation” files).

To validate *modus operandi* of SC-driven separation, we repeated the experiment using unsorted SWCNTs composed of a wider spectrum of chiralities. Also, in this case, the phase separation was found to be a function of diameter (Fig. S2, Supplementary Animation 2). The SWCNTs partitioned to the bottom phase in the ascending order of the diameter values i.e., (6,5) 0.757 nm, (8,3) 0.782 nm, (7,5) 0.829 nm, (8,4) 0.840 nm, (10,2) 0.884 nm, (7,6) 0.895 nm and (9,4) 0.916 nm in a linear fashion (Fig. S3). We also observed that the rate of partitioning is strongly dependent on the chirality of examined SWCNTs. For instance, collection of (6,5) SWCNTs from the unsorted material in the bottom phase was sudden (FWHM of 4.3 µL of SC) whereas other SWCNT species migrated over a much wider SC volume range (e.g., FWHM of (10,2) of 81.6 µL of SC or FWHM of (9,4) 86.4 µL of SC).

### Influence of non-ionic surfactant

**Figure 3:**
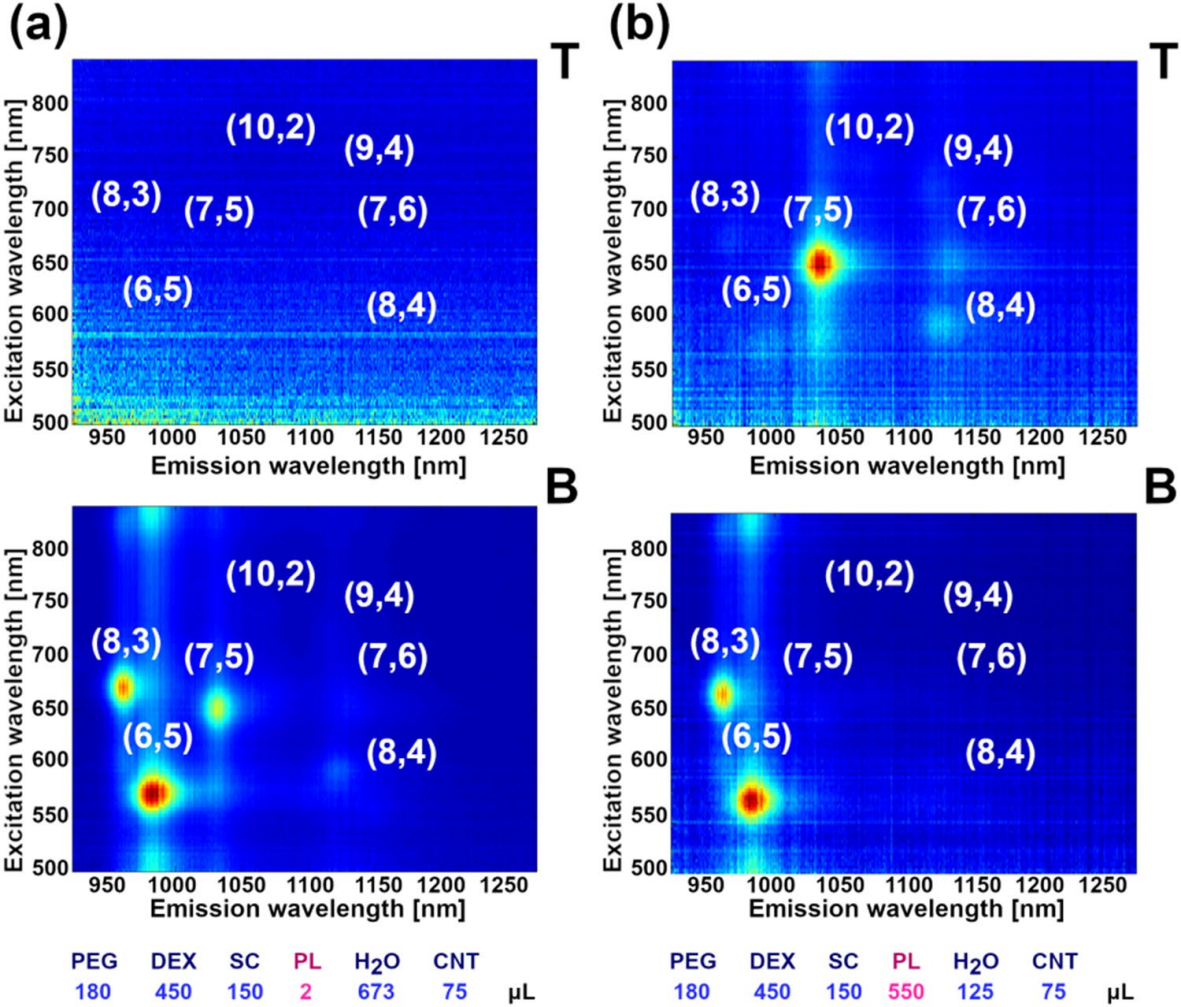
2D PLE maps of SC-dispersed, (6,5)-enriched SWCNTs sorted by ATPE upon varying the added volume of Pluronic. Example volumes: (**a**) 2 µL, and (**b**) 550 µL of PL. 2D PLE maps of remaining samples are shown in Supplementary Animation 3. Analogous data for unsorted SWCNT material is presented in Fig. S4 and Supplementary Animation 4.

We employed a tri-block co-polymer poly(ethylene glycol) (*ca. 101 units*)—poly(propylene glycol) (*ca. 56 units*)—poly(ethylene glycol) (*ca. 101 units*)^44^. This co-polymer composition is sold under the trade name of Pluronic F127. We hypothesized that its structural similarity to the PEG top phase would result in preferential occupation of this phase, which would extract the SWCNTs from the bottom. The results showed that in contrast to the action of SC moving the SWCNTs to the bottom, Pluronic resulted in SWCNTs the reverse partitioning from the bottom to the top. However, in this case, the migration started with the SWCNTs with the largest diameters (Fig. 3, Supplementary Animation 3) i.e*.*, (7,5) in the (6,5)-enriched material. Therefore, addition of aliquots of both SC and Pluronic gave rise to the isolation of a (7,5)-enriched fraction from about 250–550 µL of Pluronic.

However, when the volume of Pluronic was 300 µL or higher, movement of SWCNTs of both the (6,5) and (7,6) types to the top phase occurred (in fact, the (6,5) CNTs gradually returned to the top phase from ~ 100 µL of Pluronic, but the effect became significant above 300 µL of Pluronic). Therefore, a tradeoff between the amount of isolated (7,5) SWCNTs and the fraction purity must be established. Nevertheless, the resulting sample even at 550 µL of Pluronic was (7,5) enriched, despite the contamination with other chiralities (Fig. 3b). When the unsorted SWCNT material was subjected to such a separation routine, we received confirmation that the SWCNTs gradually relocated to the top phase in the same fashion. Species of even larger diameters such as (7,6), (8,4) and (9,4) were detected in the top phase in the first few fractions (Fig. S4, Supplementary Animation 4).

**Figure 4:**
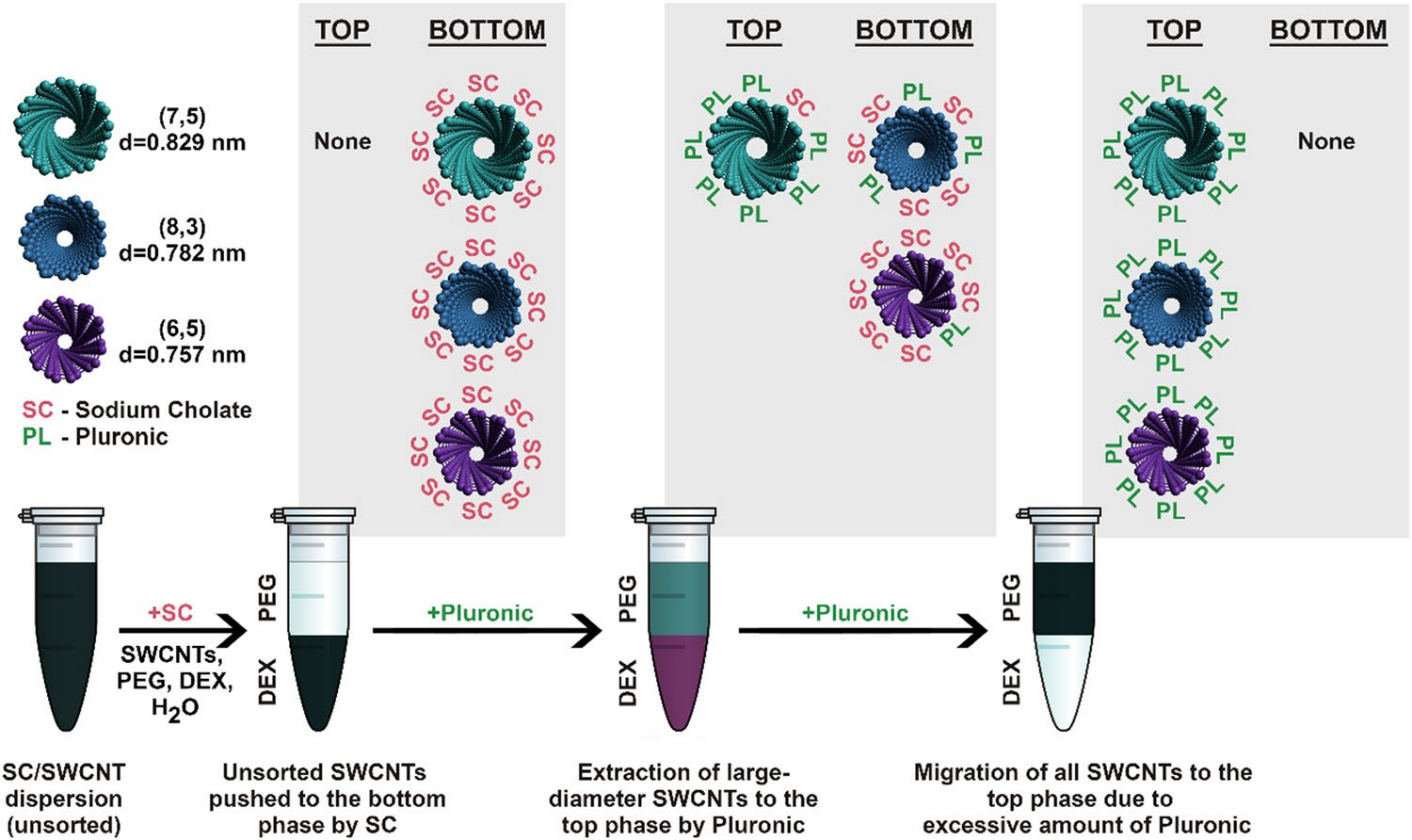
Differentiation of SC-dispersed, (6,5)-enriched SWCNTs sorted by ATPE upon varying the added volume of Pluronic in the presence of additional SC. SWCNTs were modeled by Avogadro: an open-source molecular builder and visualization tool^50^.

We gained several insights from this exercise. Primarily, to execute Pluronic-driven ATPE of SWCNTs, sufficient SC was necessary to effect phase separation. In the absence of this surfactant, the propensity of Pluronic to partition SWCNTs to the top phase was impossible, as the SWCNTs already resided in the top phase (Fig. S5, Supplementary Animation 5). Working with two surfactants makes the situation complex from the theoretical point of view because these chemical species likely form hybrid micelles. A mixed micelle system composed of Pluronic and a bile salt surfactant is expected to differ significantly in terms of core size, shape, aggregation number, etc*.* as the relative amount of one component to the other is changed^45–47^. Reports show that with the increase of SC concentration, the core of the micelles formed with Pluronic F127 becomes more hydrophobic, therefore their corona becomes more hydrophilic^48,49^. These insights are in accordance with our observations detailed above. The change of [Pluronic]/[SC] ratio as a result of the increase of [Pluronic] or [SC] shifts the encapsulated SWCNTs to the top or to the bottom phase as the SWCNT vectors become more hydrophobic or hydrophilic, respectively. A simplified mechanism of the separation process based on these conclusions is illustrated below (Fig. 4).

It has to be noted that the outcome of the separation could also be affected by varying the MW of the polymer. Lyu et al. reported that by changing the MW of the employed PEG different partitioning can be obtained^51^. The impact of Pluronic MW and its composition on the course of the separation will be studied by us in the future.

### Influence of pH

As previously reported, pH has a strong impact on the partitioning of CNTs by ATPE^38,52^. In the contribution by Flavel et al., a shift of large diameter SWCNTs to the top phase occurs upon acidification. The mechanism of that partitioning relied on the protonation-driven aggregation of sodium deoxycholate (DOC) in the DOC-SDS dispersion of SWCNTs. Gradual replacement of exposed SWCNT surface to SDS shifted the SWCNTs upwards upon SDS deposition since, as revealed before, SDS-dispersed SWCNTs prefer the top phase at high SDS concentration^42^. In our set of experiments, we tuned the amount of introduced 0.1 M HCl and 0.1 M NaOH from 0 to 20 µL to see the influence of pH on the separation of SWCNTs dispersed by SC.

**Figure 5:**
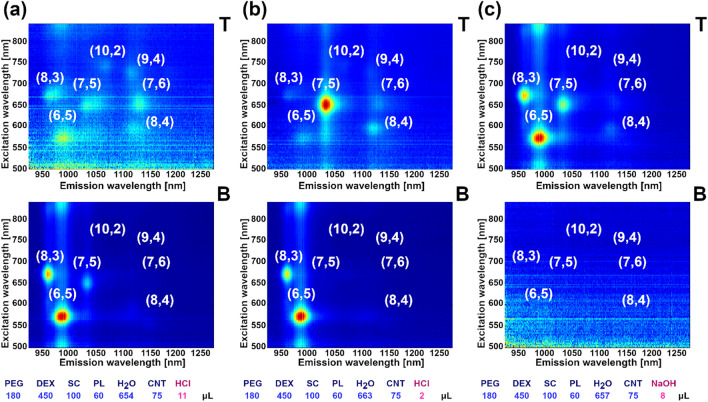
2D PLE maps of SC-dispersed, (6,5)-enriched SWCNTs sorted by ATPE upon varying pH conditions. pH was modulated using: (**a**) 11 µL HCl, (**b**) 2 µL HCl and (**c**) 8 µL NaOH. 2D PLE maps of remaining samples are shown in Supplementary Animation 6. Analogous data for unsorted SWCNTs is given in Fig. S6 and Supplementary Animation 7.

We similarly investigated the effect of pH on separation. From our results on (6,5)-enriched material (Fig. 5, Supplementary Animation 6), under the explored conditions, (7,5) SWCNTs partitioned to top phase at low pH (Fig. 5b). On the other hand, an increase in pH by the addition of NaOH, gradually partitioned the SWCNTs of smaller diameter to the top phase—first those of (8,3) type and then immediately the smaller (6,5) species (Fig. 5c). The exercise, carried out using unsorted CNT material, corroborates these findings (Fig. S6, Supplementary Animation 7). The partitioning to the top phase occurred in the descending diameter order: (7,6), (7,5), (8,3), (6,5) as the pH was increased. Evidently, the previously reported mechanism is not valid in our parameter space. This may have been due to the choice of surfactant, as we used a trihydroxy bile acid salt SC in contrast to bihydroxy DOC employed by the authors^38^. SC, in contrast to DOC, does not aggregate at low pH to form micelles of higher order^53–55^. Below pH 6.0, we expect to see first signs of protonation of SC which gives less soluble cholic acid (CA, pKa of 4.98).

At this pH, about 1 in 10 SC molecules is in the form of undissociated CA. Therefore, when the pH decreases further, more SC gradually strips from the SWCNT surface (when transformed to CA), and the exposed surface becomes available to Pluronic.

Once Pluronic adsorbs onto the exposed SWCNT surface, it increases the affinity of selected CNTs towards the top phase, due to affinity of Pluronic to PEG as shown above. We hypothesize that at low pH, large diameter SWCNTs are encapsulated to a larger degree with Pluronic rather than SC, which may explain why these SWCNTs preferentially move to the top phase. Bile acid salt surfactants have a demonstrated affinity towards low diameter SWCNTs^56^, so when the content of SC in the biphasic system is limited at low pH, these molecules selectively adsorb onto small diameter species, which keeps these SWCNTs in the bottom phase. On the other hand, addition of a minute amount of 0.1 M NaOH moved virtually all (8,3) and (6,5) SWCNTs to the top phase. It is important to point out that nuclease-free water used for the study is slightly acidic by default (pH ~ 6.0), thus the data points at 0 µL of either HCl or NaOH do not correspond to neutral pH. These small amounts of NaOH provoked a final shift of the smallest-diameter species to the top phase when they to overcome the mild acidic environment of the biphasic system, which was necessary for the shift. Lastly, we report that these experiments were conducted at a reduced content of SC from 150 to 100 µL, so that the SWCNTs would exhibit lower affinity to the bottom phase, which made the system more sensitive to the variations in pH. We also observed that excessive content of acid or base causes expected quenching of the photoluminescence emission from SWCNT dispersions^57^.

### Influence of the SWCNT species concentration

**Figure 6:**
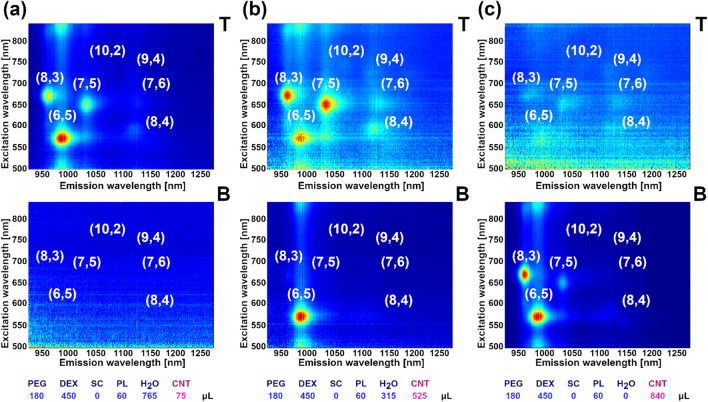
2D PLE maps of SC-dispersed, (6,5)-enriched SWCNTs sorted by ATPE upon varying concentration of the SWCNT dispersion. Example volumes: (**a**) 75 µL, (**b**) 525 µL and (**c**) 840 µL. 2D PLE maps of remaining samples are shown in Supplementary Animation 8. Analogous data for unsorted SWCNT is presented in Fig. S7 and Supplementary Animation 9.

At this point, we addressed the hypothesis that the partition coefficient of any particular SWCNT species is independent on both its own concentration and that of other species. As foreseen in an excellent review by Fagan^42^ on this topic, there may be an operational window for which thermodynamics could affect the separation course for this factor. We decided to evaluate the impact of increased SWCNT dispersion volume from 75 to 840 µL per 1.53 mL of the whole biphasic system (equivalent to SWCNT concentration change by almost an order of magnitude from 0.006 to 0.052 wt%, Fig. 6). The approach resembles the concept developed by Kataura’s group for SWCNT separation using gel chromatography^29^, who reported that various SWCNT types have a different interaction with the stationary phase, which can be exploited in the high SWCNT concentration regime. The authors reported that once the system is overloaded with SWCNTs, selective adsorption of SWCNTs of defined structure occurs in the first column while the rest of the dispersion remains in the mobile phase awaiting to be deposited in one of the subsequent columns. Adsorption of SWCNTs in such a series of columns works on a principle of decreased affinity of SWCNTs of particular structure towards the gel when all the absorption sites are occupied.

We investigated whether a solid–liquid equilibrium can be obtained similar to the solid–solid equilibrium revealed in the work by Kataura et al.^29^. We noticed that initially at a low SWCNT concentration of (6,5)-enriched CNT material, all the CNT types resided in the top phase (in the absence of SC, which would otherwise shift all of them to the bottom, vide infra). Interestingly, when the content of CNTs was gradually increased, a point was reached at which low-diameter SWCNTs crossed the interface and entered the bottom DEX phase (Fig. 6, Supplementary Animation 8). When the volume of SWCNT dispersion was increased seven-fold from 75 (Fig. 6a) to 525 µL of the parent dispersion, SWCNTs of the (6,5) chirality were clearly identified in the bottom (Fig. 6b). Further increases to the quantity of SWCNTs eventually resulted in migration of other SWCNT types as (8,3) and (7,5) from the top (Fig. 6c). Again, the emergence of these species therein was rapid and not gradual.

Therefore, based on these findings, we see that the SWCNTs are not simply partitioning from the top phase in the linear way as a function of SWCNT content, but that a certain concentration threshold must be established to observe the phenomenon. An appropriate SWCNT concentration of a particular type must be reached so that their dispersion in the bottom phase would become preferential rather than in the top, by default. The shift may also be promoted by the gradually increased amount of SC in the ATPE system, present on the surface of SWCNTs making them dispersible in the aqueous medium. Although these species are likely not liberated from the SWCNT surface during the described processing, it cannot be excluded that they can affect the hydration level of both the phases to some extent. Lastly, the enclosed PLE excitations-emission maps show that a surplus of SWCNT dispersion in the PEG phase results in SWCNT aggregation (Fig. 6c), which quenches the PLE^58^. This effect is not observed for the bottom phase because only a small fraction of the starting CNT mixture shifts to the dextran matrix.

As a next step, we used the unsorted material to probe the mechanism of this phenomenon (Fig. S7, Supplementary Animation 9). Once again, we observed a clear diameter dependence on concentration-mediated partitioning, as CNTs relocated to the bottom phase. Complete transition of (6,5) and (8,3) SWCNT species was witnessed, while for the rest of the available chiralities, only the beginning of the shift can be seen. The employed SWCNT volume could not be increased further as there was no remaining H_2_O, which could be substituted. For all the experiments, the volume of the ATPE system was kept constant and the components were introduced at the expense of additional water. Nevertheless, based on the data, one could extrapolate the conditions under which such a shift should take place for the analyzed SWCNT chiralities present in the investigated unsorted material.

The observed trends anticipate poor selectivity for the larger-diameter SWCNT species, as their transition conditions are very much overlapping. We take note of the results that occurred when the separation of CNTs was conducted in the presence of SC while overloading the biphasic system with CNTs (Fig. S8, Supplementary Animation 10). As indicated before, under such conditions, the CNTs reside in the bottom phase by default. An attempt to cause a similar distribution of CNTs, between top and bottom phases, by significantly increasing the concentration of CNTs, did not work in the same way. No CNTs were detected in the top phase regardless of the introduced volume of the parent dispersion. This result, however, validates the proposed mechanism given above, that a surplus of CNTs in the biphasic system does not merely cause partition to the opposite phase, but there is also a certain selectivity, which can be reached upon introduction of an excessive amount of CNTs.

### Separation of DNA-dispersed CNTs

Surfactant-wrapped CNTs are often unsuitable for certain applications, such as in biology, due to high concentration of dispersing agents in the solution^59^. As a consequence, SWCNT hybrids are often re-dispersed with non-ionic surfactants^60,61^ or DNA^62^. However, re-dispersion often produces low yields and may be labor-intensive^63,64^. We wanted to evaluate whether the ATPE system can be optimized, using high-throughput analysis, to non-ionic surfactant and DNA-SWCNT hybrids. For this experiment, we selected a TTT-CCC-TTT-CCC-CCC ssDNA super-sequence recently reported to have a high affinity towards (8,3) and a few other SWCNT types in ATPE^51^. A super-sequence, according to this work, is a sequence able to resolve more than two kinds of SWCNTs from a single DNA-SWCNT dispersion.

**Figure 7:**
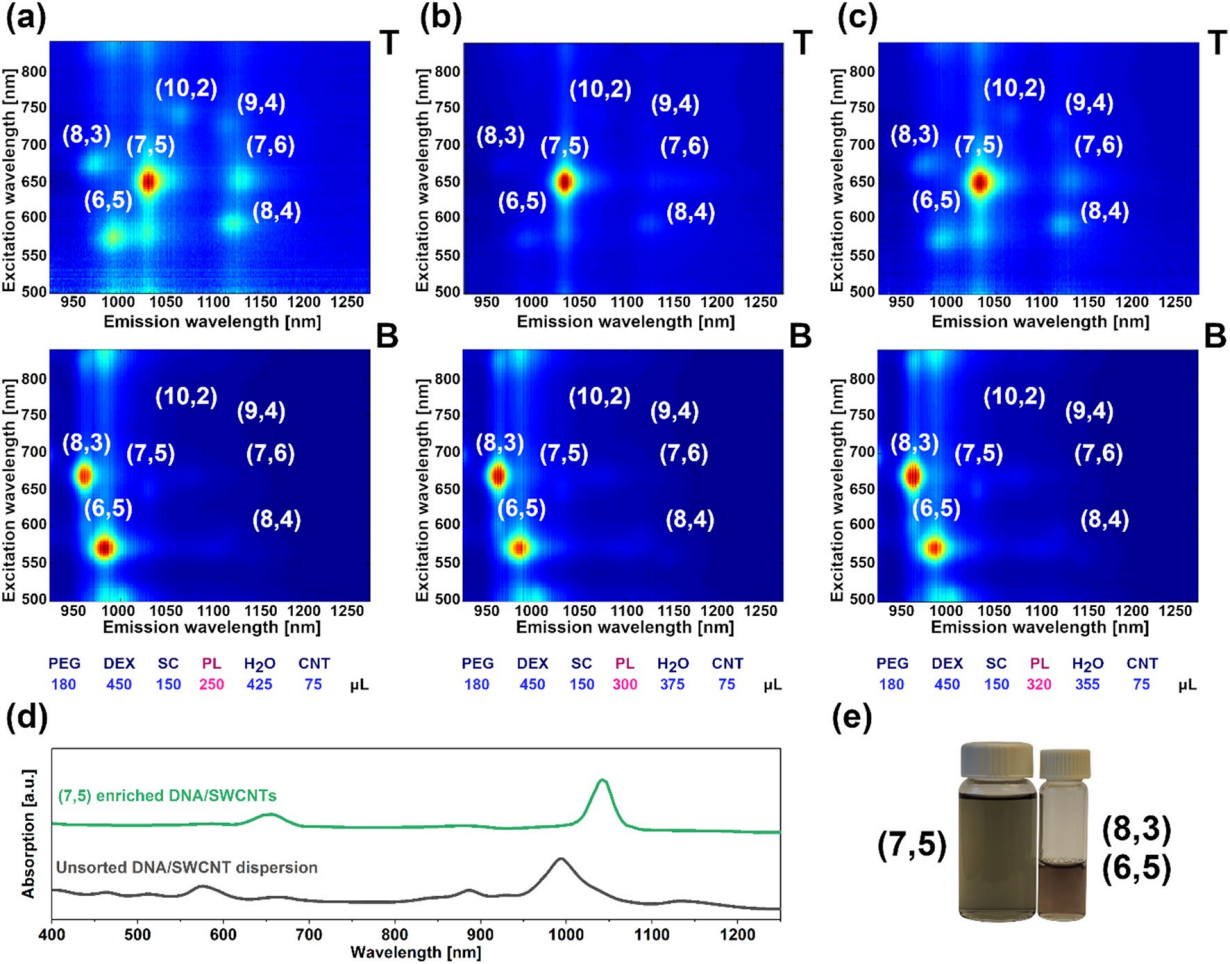
Isolation of DNA-dispersed SWCNTs. 2D PLE maps of DNA-dispersed, (6,5)-enriched SWCNTs sorted by ATPE upon varying the volume of Pluronic to (**a**) 250 µL, (**b**) 300 µL and (**c**) 320 µL. (**d**) Absorption spectra of (7,5) rich SWCNT phase and the parent dispersion, (**e**) vials with isolated chirality-enriched SWCNT dispersions (both from the samples obtained at the content of Pluronic equal to 300 µL).

To our delight, we observed that adjustment of the Pluronic and SC content in the biphasic system resulted in separation of (7,5) SWCNTs with remarkable purity in a single step (Fig. 7b). It is clear that the optimum volume of Pluronic was 300 µL at 150 µL of SC, similar to the parameters established for SC-dispersed SWCNTs, to isolate the (7,5) chirality. Lower (250 µL, Fig. 7a) and higher (320 µL, Fig. 7c) Pluronic content led to the emergence of a variety of other SWCNT chiralities in the top phase. For an intermediate volume of Pluronic (300 µL), the top phase almost exclusively consists of SWCNTs of the (7,5) chirality. The bottom phase, on the other hand, was comprised of (6,5) and (8,3) SWCNTs regardless of the Pluronic volume. The (8,3) SWCNT content in the bottom phase was notably larger as compared with the results from sorting surfactant-dispersed SWCNTs due to the documented compatibility of the selected DNA super-sequence with e.g. (8,3) SWCNTs.

The absorption spectra collected after the introduction of 300 µL of Pluronic confirmed the results of the 2D PLE mapping, as SWCNTs of (7,5) and (8,3) + (6,5) type were chiefly detected in the top and bottom phases, respectively (Fig. 7d). The colors of the dispersions were also visibly evident (Fig. 7e). The dispersion with (7,5) SWCNTs was teal whereas the mixture of (6,5) (normally violet) and (8,3) (typically cyan) gave a violet purple hue, as in other reports on SWCNT separation. Finally, a Raman spectrum of the sorted material revealed high purity of the (7,5)-enriched SWCNT fraction (Fig. S9).

It appears that the origin of the successful separation in this experiment stems from establishing appropriate ATPE conditions rather than any specificity potentially offered by the selected ssDNA sequence, which exhibits aforementioned affinity towards (8,3) CNTs. (7,5) CNTs were again isolated in the top phase analogously to the case when SC was the dispersant (Fig. 3) and Pluronic was the partitioning modulator as well. Therefore, under the explored separation conditions, the dominant factor enabling the successful separation is the action of Pluronic. Due to its preferential deposition onto large diameter CNTs, when using ssDNA-dispersed CNTs, one can produce chirality-defined CNTs of improved biocompatibility, simultaneously avoiding the laborious redispersion routines.

## Conclusions

In summary, we demonstrated that the resolution and the facility of the ATPE method for separation of surfactant-dispersed SWCNTs can be notably increased upon the introduction of a non-ionic tri-block copolymer surfactant into the system. When combined with an appropriate amount of ionic surfactant, one can direct the SWCNT partitioning in the desired way. Consequently, fractions of satisfactory chiral purity of (6,5) or (7,5) SWCNTs can be obtained in a single step. We also noticed that it is possible amend the procedure to separate DNA-encapsulated SWCNTs, potentially facilitating downstream applications. As a result, (7,5) SWCNTs with notable spectral homogeneity were obtained, which do not require re-wrapping. Other effects on separation such as those mediated by pH and SWCNT concentration were also studied in detail. Interestingly, overloading of the biphasic system with SWCNTs enabled preferential isolation of the smallest SWCNT diameters from the material.

Improved metrology methods, such as high-throughput photoluminescence excitation-emission plots, employed throughout processes, may potentially enable researchers to better understand and improve separation methods. Ideally, the measurement setup should be coupled with an extension measuring absorption spectra since this analysis type is less susceptible to SWCNT’s surroundings. This would also directly eliminate the influence of PL quantum yield, which is a function of chirality, from consideration. In the present study, we had to employ appropriate correction factors to remove this possible source of bias.

Straightforward methods for harvesting SWCNTs of particular electronic and optical characteristics are required to implement them widely. Currently, one of the key limitations is the necessity to employ demanding separation methods that have not been adopted by industry or many research labs. The proposed protocol based on the simple ATPE approach constitutes an opportunity to expand the use of chirality-defined SWCNT species.

## Supplementary Information

Supplementary Figures.

Supplementary Information 1.

Supplementary Information 2.

Supplementary Information 3.

Supplementary Information 4.

Supplementary Information 5.

Supplementary Information 6.

Supplementary Information 7.

Supplementary Information 8.

Supplementary Information 9.

Supplementary Information 10.
