# Supplementary material for: En route to single-step, two-phase purification of carbon nanotubes facilitated by high-throughput spectroscopy
Source: Sci Rep. 2021 May 19;11:10618. doi: 10.1038/s41598-021-89839-4 (PMC8134628; doi:10.1038/s41598-021-89839-4)
Supplement: Supplementary file 1 — Supplementary Figures. [file 41598_2021_89839_MOESM1_ESM.docx]

Supplementary Information file

**En route to single-step, two-phase purification of carbon nanotubes facilitated by high-throughput spectroscopy**

Blazej Podlesny^a^, Barbara Olszewska^a^, Zvi Yaari^b,c^, Prakrit V. Jena^b,c^, Gregory Ghahramani^b,c^,
Ron Feiner^b,c^, Daniel A. Heller^b,c,*^, Dawid Janas^a,*^

*^a^ Department of Organic Chemistry, Bioorganic Chemistry and Biotechnology, Silesian University of Technology, B. Krzywoustego 4, 44-100 Gliwice, Poland*

*^b^ Molecular Pharmacology Program, Memorial Sloan Kettering Cancer Center, New York, NY, USA*

*^c^ Department of Pharmacology, Weill Cornell Medicine, New York, NY, USA*

*Corresponding authors: [HellerD@mskcc.org](mailto:HellerD@mskcc.org) (D.A.H.) and [Dawid.Janas@polsl.pl](mailto:Dawid.Janas@polsl.pl) (D.J.)

The intensities of spectral features of all detected CNT chiralities in both top and bottom phases were recorded and subjected to correction for the excitation and acquisition aberrations introduction by the employed apparatus. Intensity of the background was subtracted. Where possible, to follow the ATPE partitioning course, the obtained values of intensity for each chirality were added, normalized to unity, and the relative distribution of particular CNT chiralities in both the phases was calculated. Then, this distribution was studied as a function of the introduced volume of *e.g.* SC, Pluronic, NaOH or HCl. An example of such approach is given Fig. S1 below, wherein (6,5)-enriched SWCNT material was sorted by ATPE at various volumes of SC from 0 µL to 765 µL. Three CNT types were detected in the material at the selected acquisition parameters: (6,5), (8,3) and (7,5). One can see that the transition from one phase to the other (in this case from the bottom to the top) is rapid and occurs in the specified SC volume range (Fig. S1a,c,e). To study the rate of partitioning of this process first derivatives of these curves were calculated (Fig. S1b,d,f). The widths and heights of the generated curves display how sudden is the isolation of a particular CNT type under the selected conditions. The degree of separation of these curves on the other hand reveal how selective are the operational conditions. Ideally, the curves should be slender and have no areas of overlap with each other to give fractions of monochiral nature.

The results of analogous analyses conducted for other experiments are provided in the top panels of Supplementary Animation files, for which CNT signal to noise ratio was sufficient to prepare them.


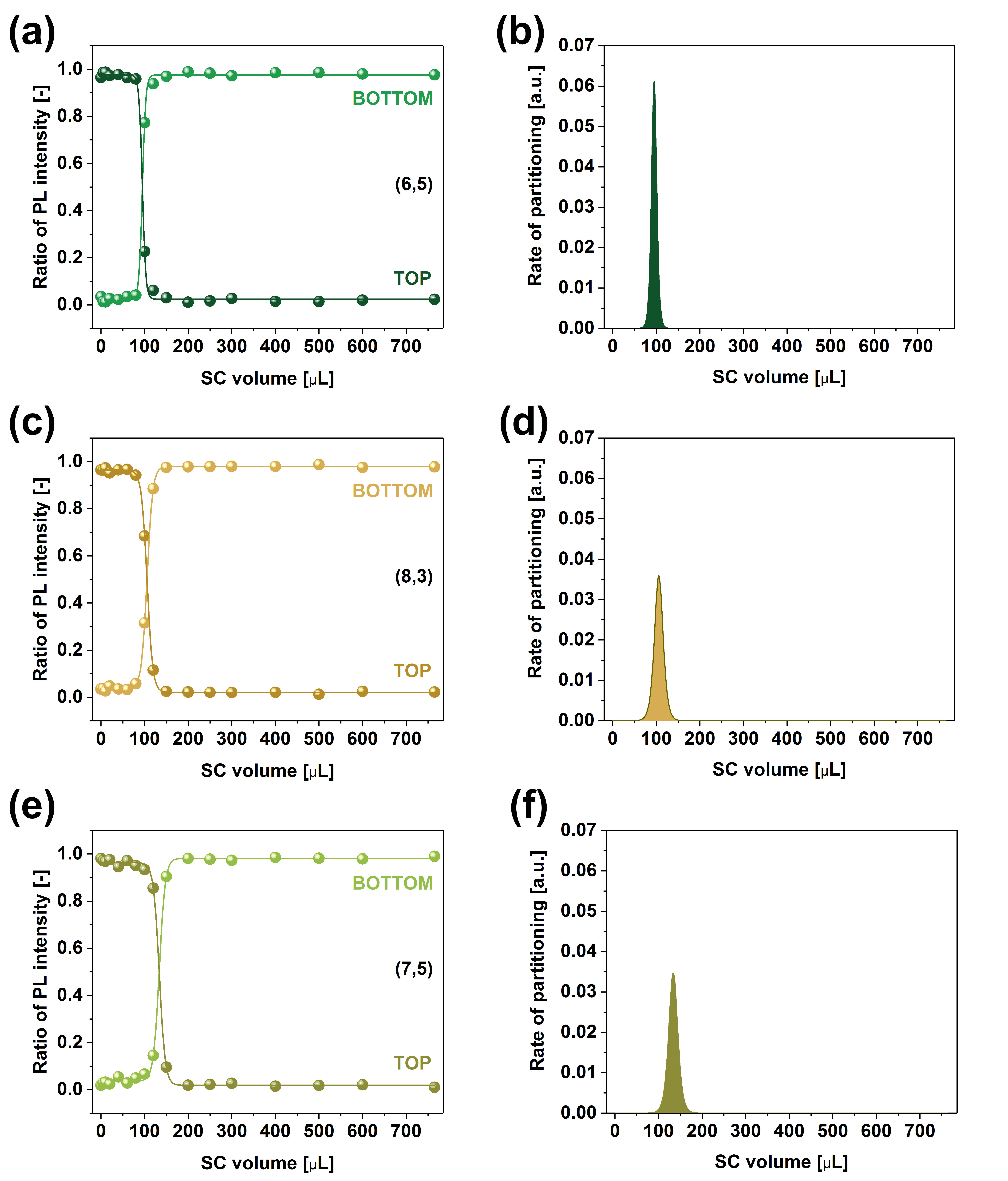


**Figure S1** (a) relative intensity of (6,5) excitation-emission features in the bottom and top phases as recorded by 2D PLE mapping for all the data points acquired as a function of SC volume, (b) corresponding first derivative plot, (c) relative intensity of (8,3) excitation-emission features in the bottom and top phases as recorded by 2D PLE mapping for all the data points acquired as a function of SC volume, (d) corresponding first derivative plot, (e) relative intensity of (7,5) excitation-emission features in the bottom and top phases as recorded by 2D PLE mapping for all the data points acquired as a function of SC volume, (f) corresponding first derivative plot. The experiment was conducted using (6,5)-enriched SWCNT material.


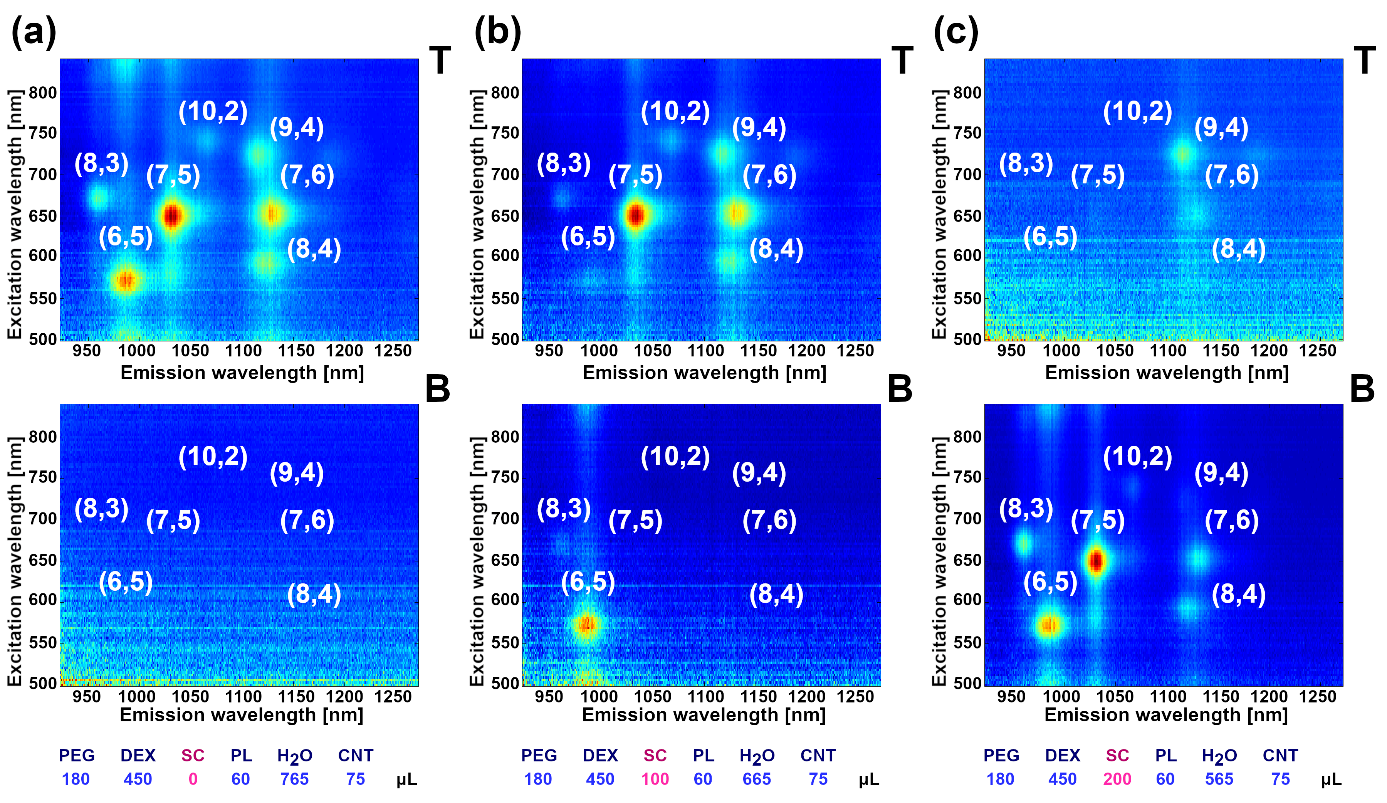


**Figure S2** 2D PLE maps of SC-dispersed, unsorted SWCNTs sorted by ATPE upon varying the added volume of SC. Example volumes: (a) 0 µL, (b) 100 µL and (c) 200 µL of SC. 2D PLE maps of remaining samples are shown in Supplementary Animation 2.


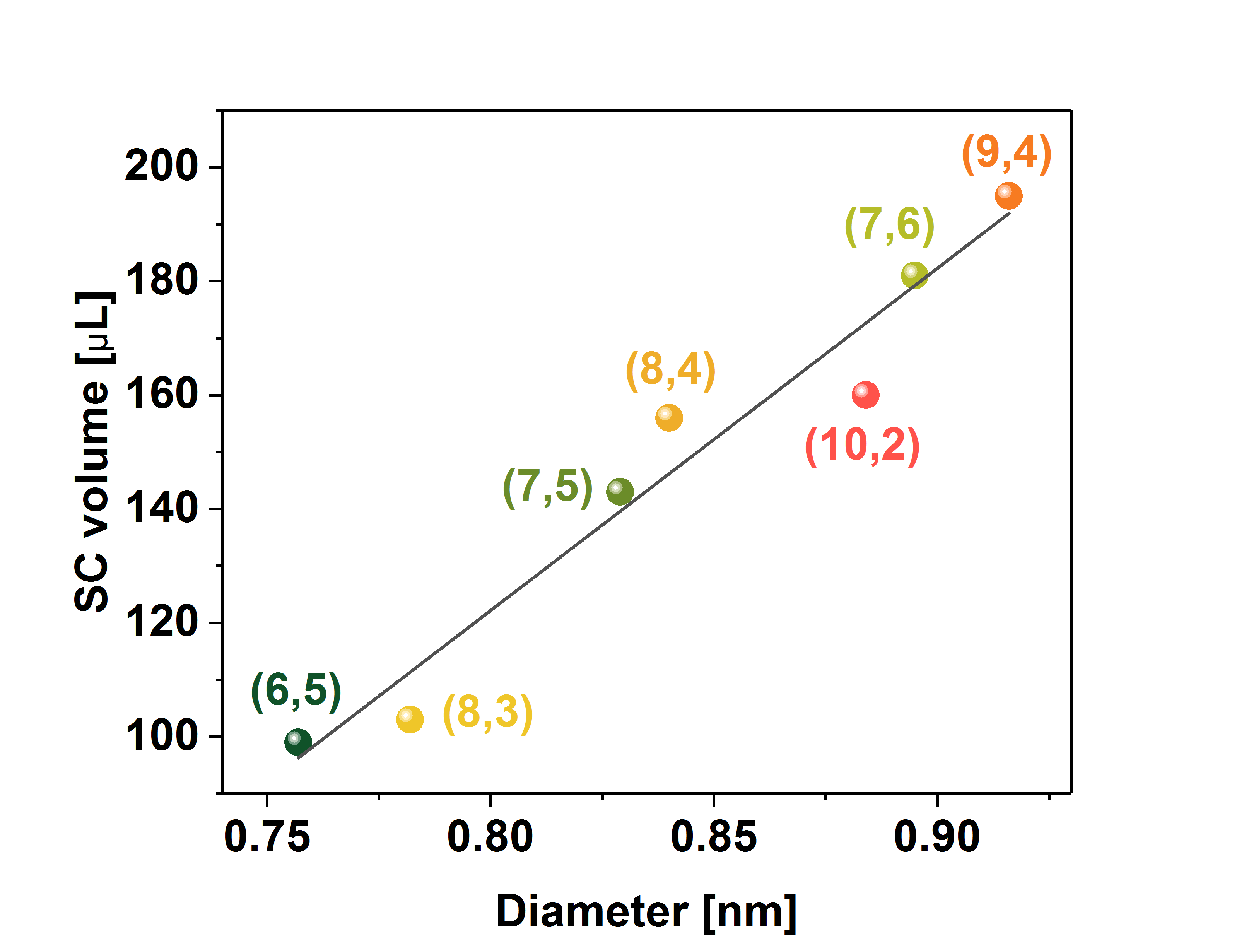


**Figure S3** The relation of the diameter of the isolated CNTs to the volume of introduced SC for unsorted SWCNTs


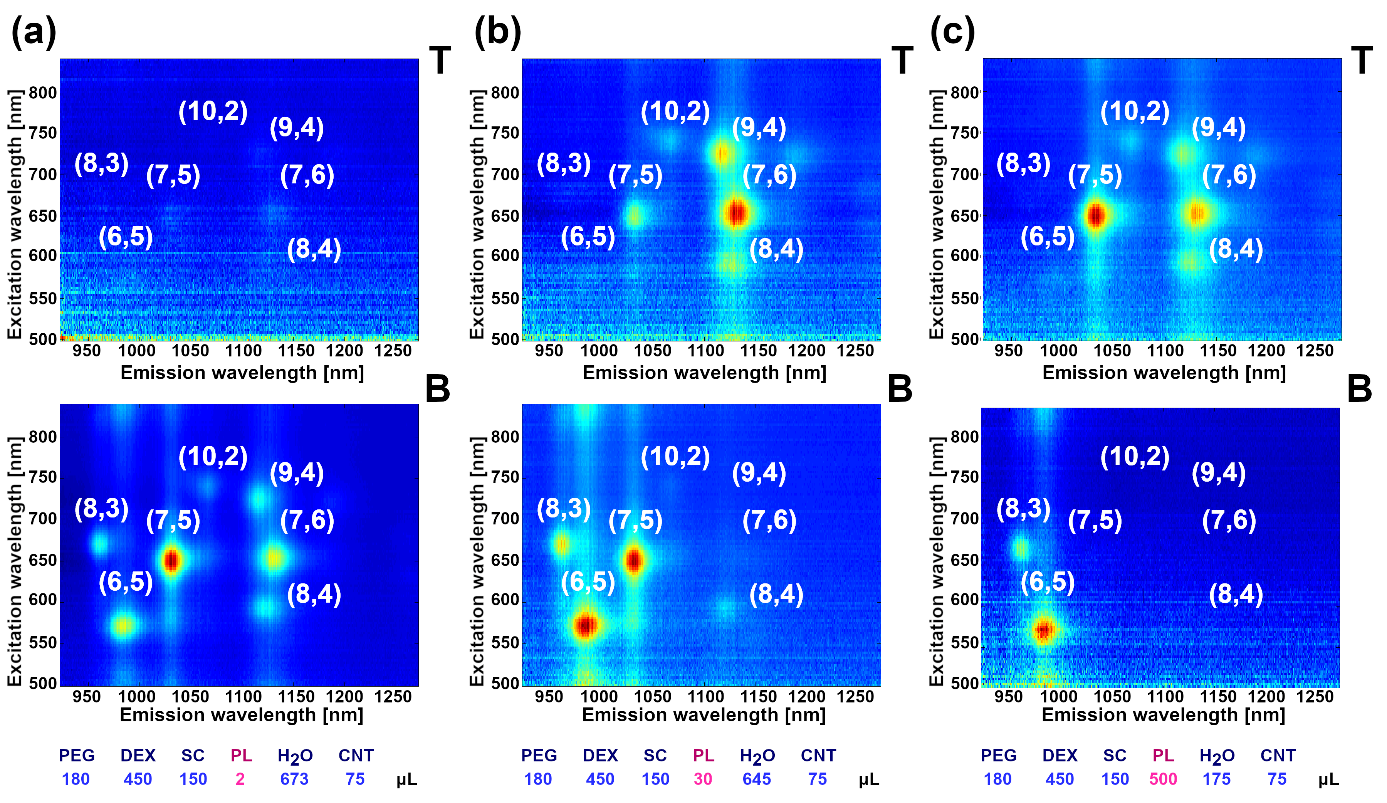


**Figure S4** 2D PLE maps of SC-dispersed unsorted SWCNTs sorted by ATPE upon varying the added volume of Pluronic. Example volumes: (a) 2 µL, (b) 30 µL (c) 500 µL of Pluronic. 2D PLE maps of remaining samples are shown in Supplementary Animation 4.


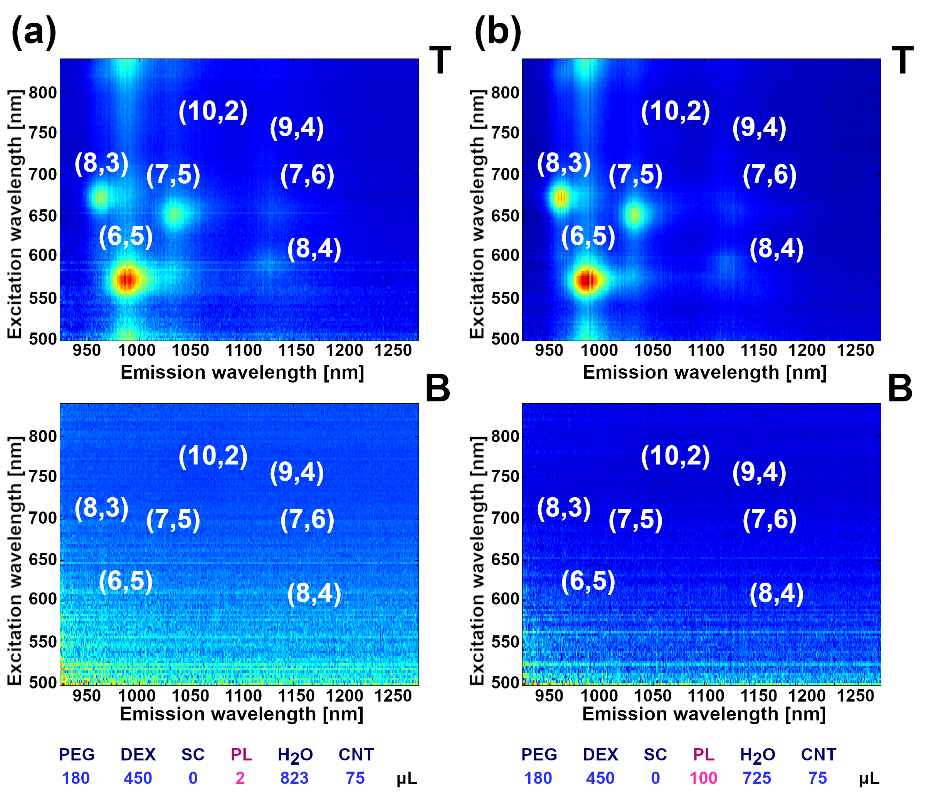


**Figure S5** 2D PLE maps of SC-dispersed, (6,5)-enriched SWCNTs sorted by ATPE upon varying the added volume of Pluronic (in the absence of SC). Example volumes: (a) 2 µL and (b) 100 µL. 2D PLE maps of remaining samples are shown in Supplementary Animation 5.


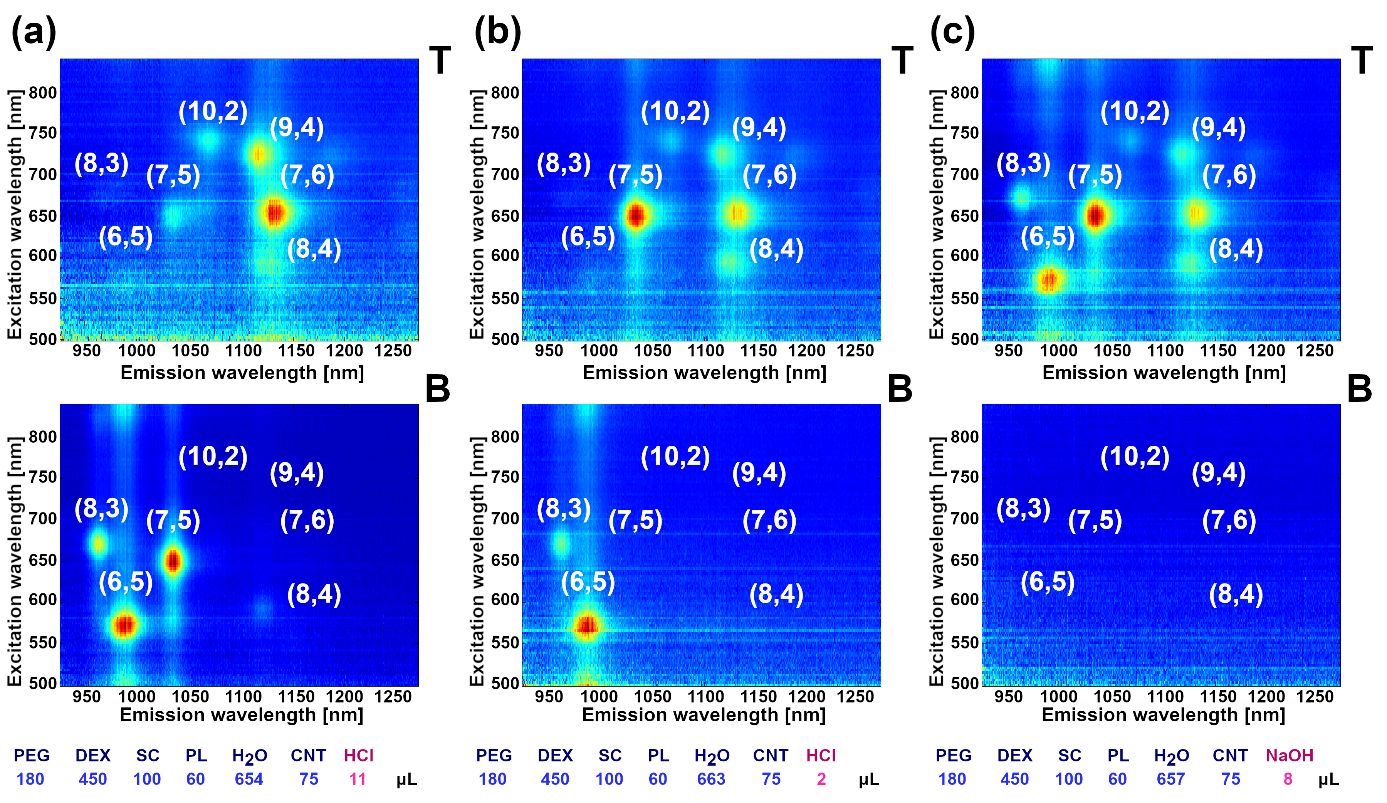


**Figure S6** 2D PLE maps of SC-dispersed unsorted SWCNTs sorted by ATPE upon varying pH conditions. pH was modulated using: (a) 11 µL HCl, (b) 2 µL HCl and (c) 8 µL NaOH. 2D PLE maps of remaining samples are shown in Supplementary Animation 7.


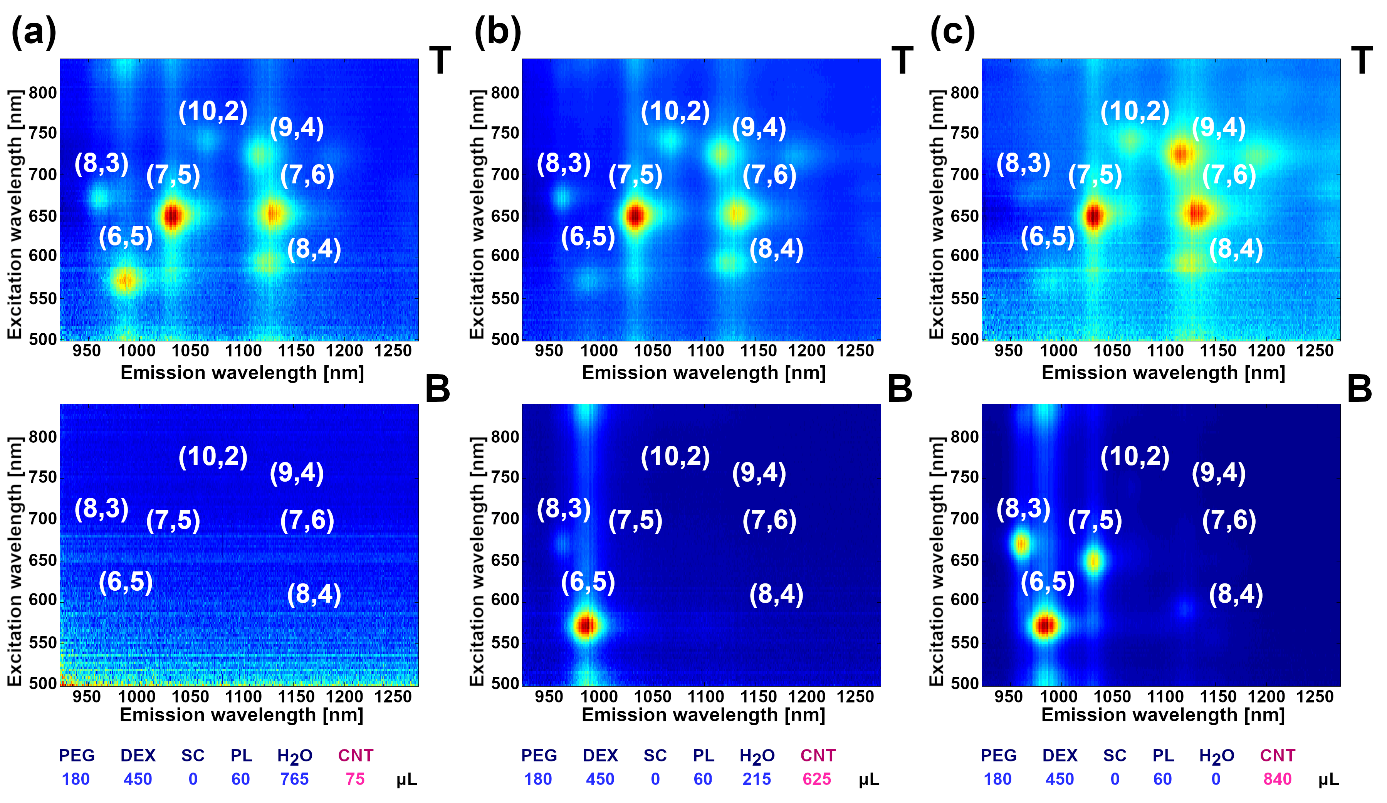


**Figure S7** 2D PLE maps of SC-dispersed unsorted SWCNTs sorted by ATPE upon varying concentration of CNT dispersion. Example volumes: (a) 75 µL, (b) 625 µL and (c) 840 µL. 2D PLE maps of remaining samples are shown in Supplementary Animation 9.


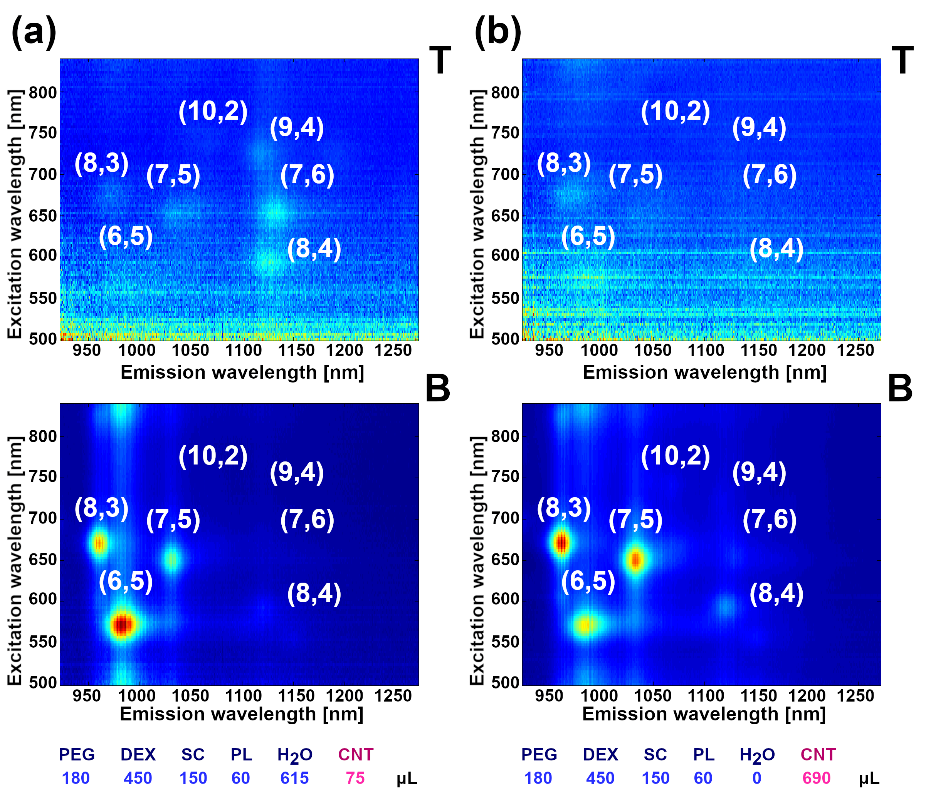


**Figure S8** 2D PLE maps of SC-dispersed, (6,5)-enriched SWCNTs sorted by ATPE upon varying concentration of CNT dispersion (in the presence of SC). Example volumes: (a) 75 µL and (b) 690 µL. 2D PLE maps of remaining samples are shown in Supplementary Animation 10.


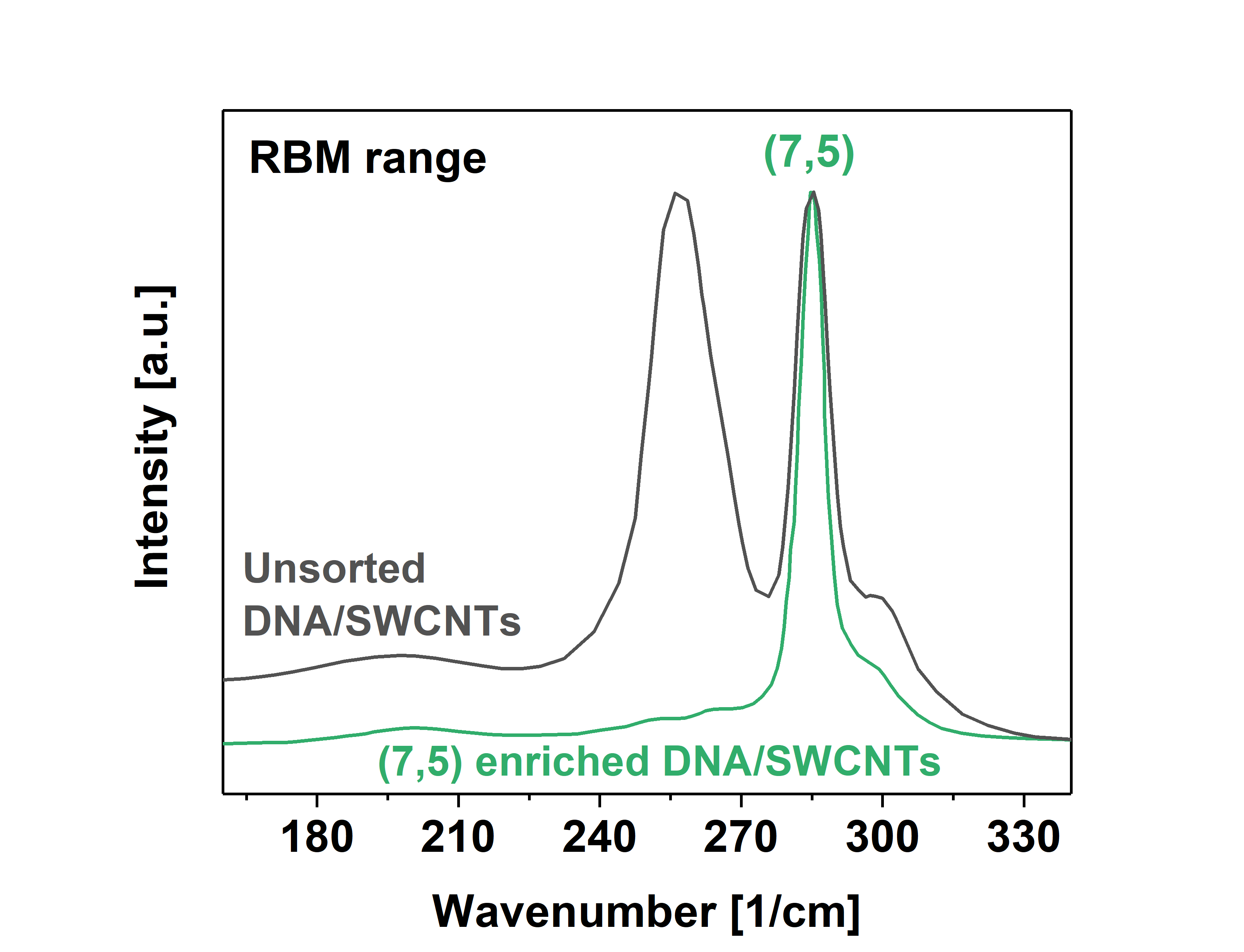


**Figure S9** Raman spectra of unsorted and (7,5) enriched SWCNTs dispersed with ssDNA after the ATPE separation.
