# Supplementary figures and images for: En route to single-step, two-phase purification of carbon nanotubes facilitated by high-throughput spectroscopy
Source: Sci Rep. 2021 May 19;11:10618. doi: 10.1038/s41598-021-89839-4 (PMC8134628; doi:10.1038/s41598-021-89839-4)

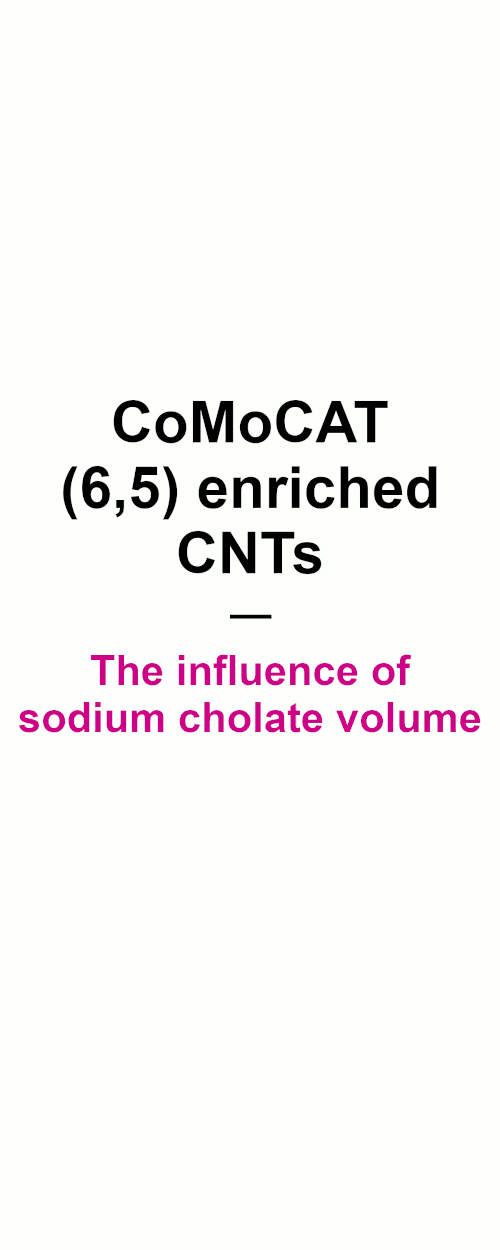

Supplement: Supplementary file 2 — Supplementary Information 1. [file 41598_2021_89839_MOESM2_ESM.gif]

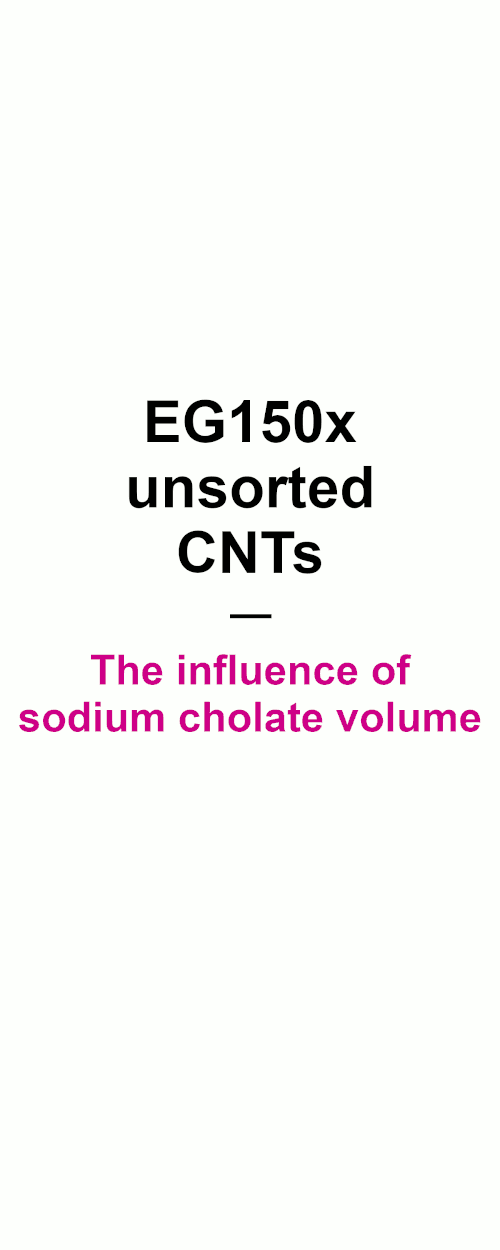

Supplement: Supplementary file 3 — Supplementary Information 2. [file 41598_2021_89839_MOESM3_ESM.gif]

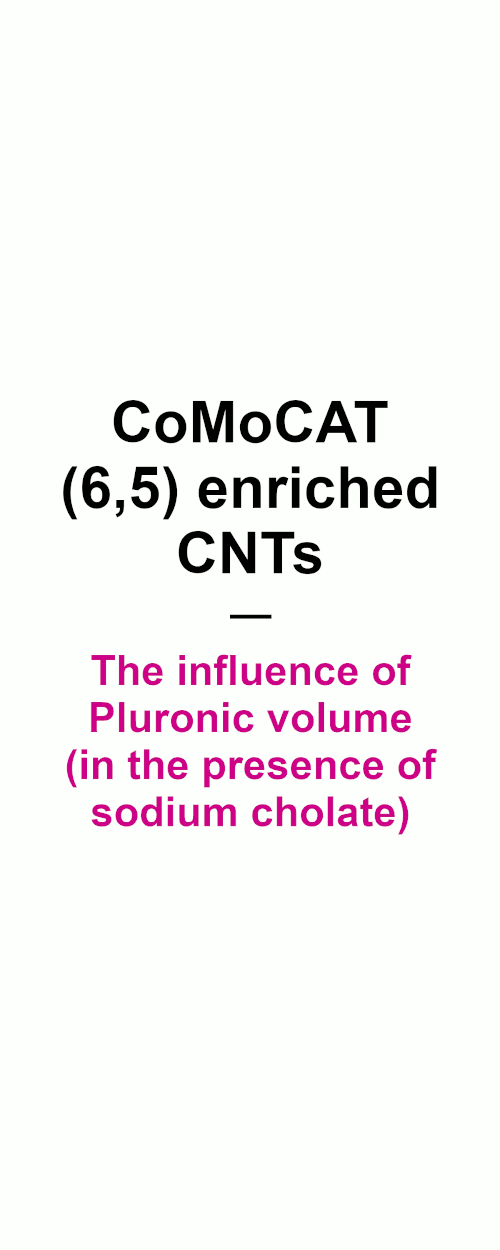

Supplement: Supplementary file 4 — Supplementary Information 3. [file 41598_2021_89839_MOESM4_ESM.gif]

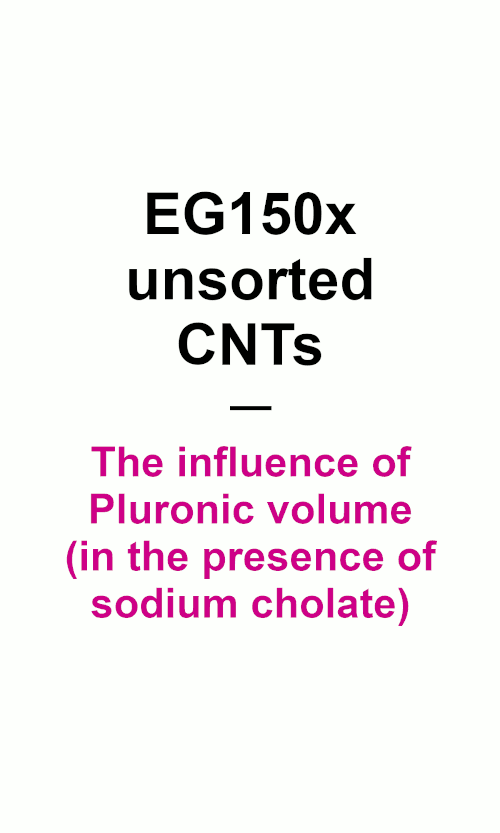

Supplement: Supplementary file 5 — Supplementary Information 4. [file 41598_2021_89839_MOESM5_ESM.gif]

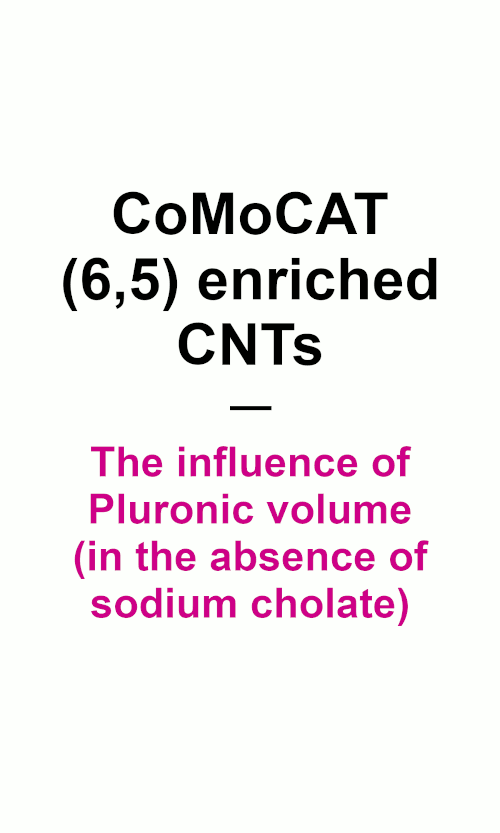

Supplement: Supplementary file 6 — Supplementary Information 5. [file 41598_2021_89839_MOESM6_ESM.gif]

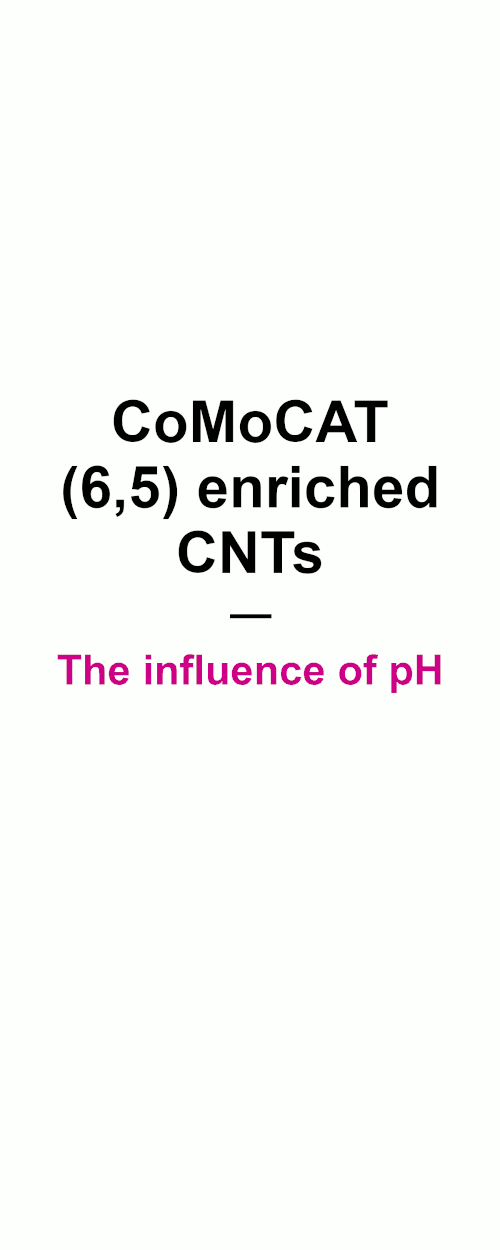

Supplement: Supplementary file 7 — Supplementary Information 6. [file 41598_2021_89839_MOESM7_ESM.gif]

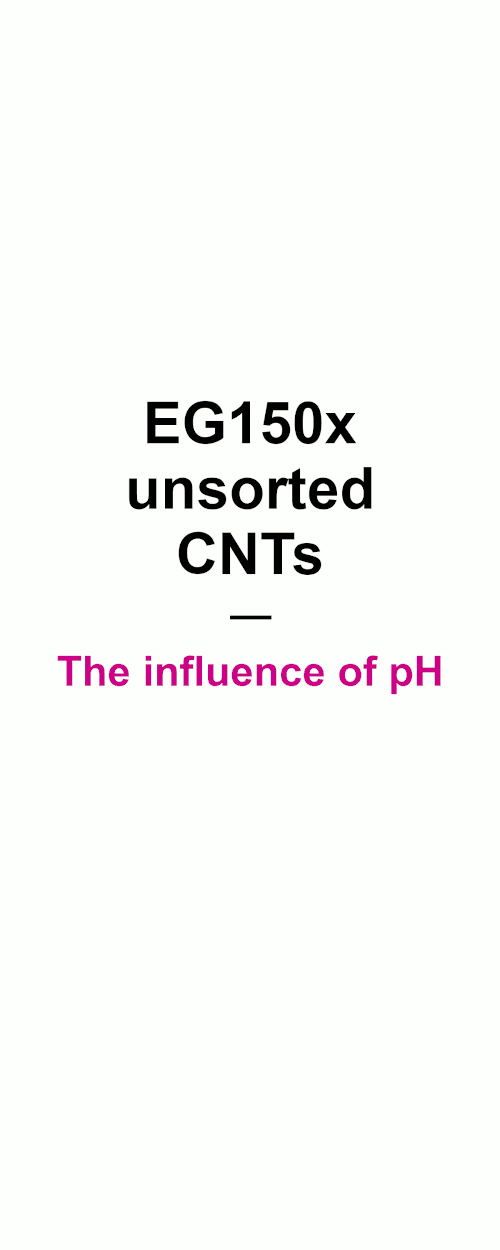

Supplement: Supplementary file 8 — Supplementary Information 7. [file 41598_2021_89839_MOESM8_ESM.gif]

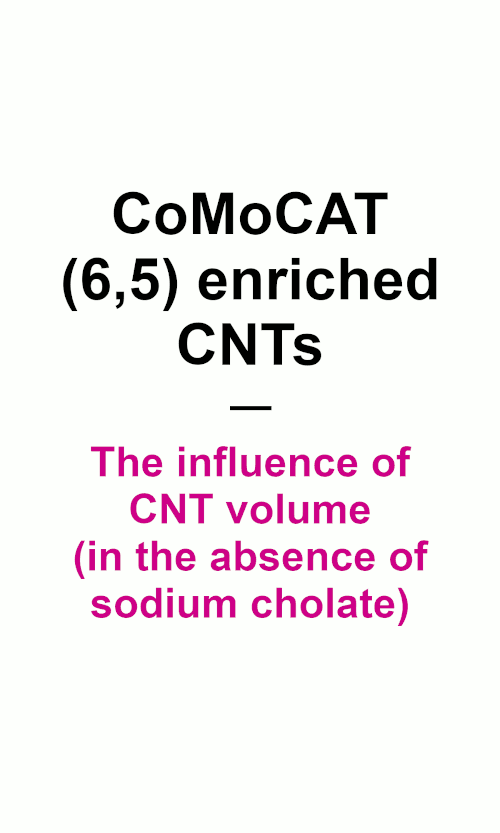

Supplement: Supplementary file 9 — Supplementary Information 8. [file 41598_2021_89839_MOESM9_ESM.gif]

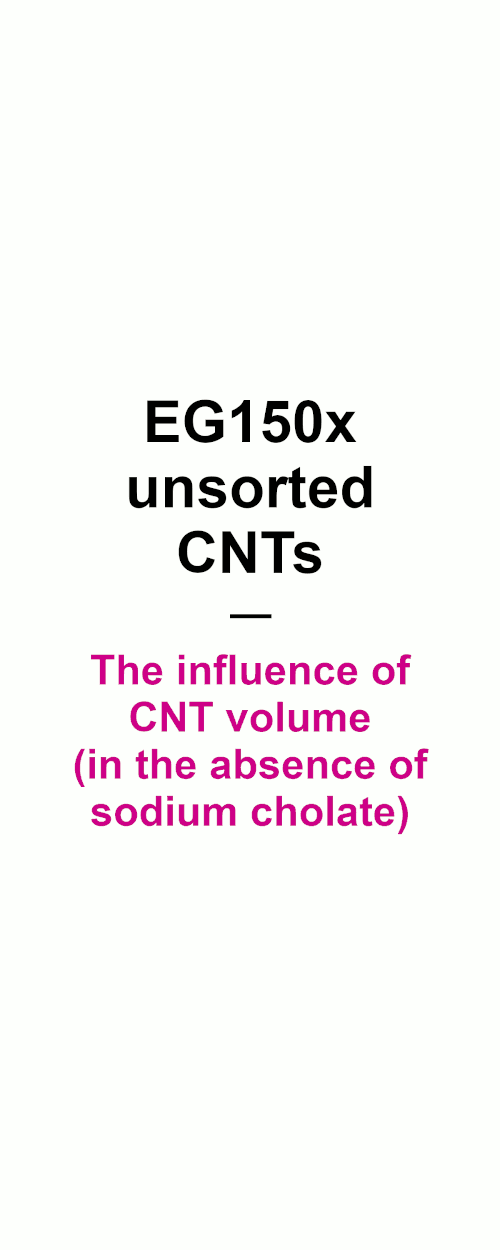

Supplement: Supplementary file 10 — Supplementary Information 9. [file 41598_2021_89839_MOESM10_ESM.gif]

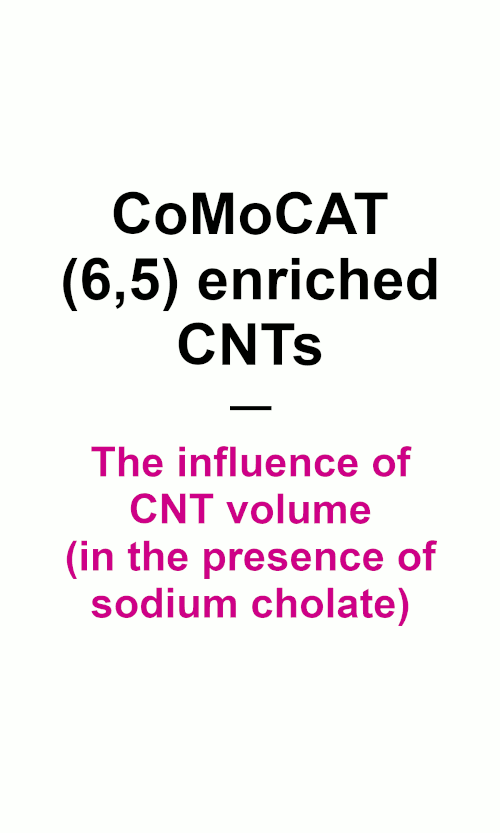

Supplement: Supplementary file 11 — Supplementary Information 10. [file 41598_2021_89839_MOESM11_ESM.gif]
